# Application of Echocardiography on Transgenic Mice with Cardiomyopathies

**DOI:** 10.1155/2012/715197

**Published:** 2012-05-21

**Authors:** G. Chen, Y. Li, J. Tian, L. Zhang, P. Jean-Charles, N. Gobara, C. Nan, J.-P. Jin, X. P. Huang

**Affiliations:** ^1^Department of Cardiology, The Children's Hospital of Chongqing Medical University, Chongqing 400014, China; ^2^Department of Biomedical Science, College of Medicine, Florida Atlantic University, Boca Raton, FL 33431, USA; ^3^Department of Physiology, Wayne State University College of Medicine, Detroit, MI 48201, USA

## Abstract

Cardiomyopathies are common cardiac disorders that primarily affect cardiac muscle resulting in cardiac dysfunction and heart failure. Transgenic mouse disease models have been developed to investigate the cellular mechanisms underlying heart failure and sudden cardiac death observed in cardiomyopathy cases and to explore the therapeutic outcomes in experimental animals in vivo. Echocardiography is an essential diagnostic tool for accurate and noninvasive assessment of cardiac structure and function in experimental animals. Our laboratory has been among the first to apply high-frequency research echocardiography on transgenic mice with cardiomyopathies. In this work, we have summarized our and other studies on assessment of systolic and diastolic dysfunction using conventional echocardiography, pulsed Doppler, and tissue Doppler imaging in transgenic mice with various cardiomyopathies. Estimation of embryonic mouse hearts has been performed as well using this high-resolution echocardiography. Some technical considerations in mouse echocardiography have also been discussed.

## 1. Introduction

Cardiomyopathies have been considered to represent diseases that primarily affect cardiac muscle. On the ground of their morphology and pathophysiology, cardiomyopathy may be classified into three major types: hypertrophic cardiomyopathy (HCM), dilated cardiomyopathy (DCM), and restrictive cardiomyopathy (RCM). In the past, most cardiomyopathy cases were described as idiopathic cardiomyopathies, that is, etiology is unknown [1, 2]. Now, we know that most cardiomyopathy cases are heritable [3] and several mutations in cardiac muscle genes, such as myosin, tropomyosin, cardiac troponin T (cTnT), and cardiac troponin I (cTnI) have been identified to be associated with cardiomyopathies [4–7].

Clinical studies showed great heterogeneity among the genetic cardiomyopathic patients even when they carry the same mutation [8–11]. The biggest challenge is that no data are available so far to confirm the overall expression and the incorporation rate of the mutant sarcomeric proteins in the heart. Furthermore, other environmental and genetic factors may also contribute to the heterogeneity of the disease manifestation. Recently, transgenic animal models have been generated to mimic various cardiomyopathies by expressing the mutant cardiac myofibril proteins observed in human patients [12, 13]. Studies with transgenic animals will fill the gap in cardiomyopathy research between the *in vitro* assays using reconstituted myofibrils and the clinical studies with the patients carrying the mutant genes, which will undoubtedly improve our understanding of the mechanisms underlying the cardiomyopathies and provide us with clues for the prevention and treatment of the diseases.

In general, in the case of DCM, heart failure is characterized by systolic dysfunction (i.e., reduced ejection fraction), whereas HCM and RCM are characterized by diastolic dysfunction (i.e., impaired relaxation) [14]. To evaluate the phenotypes, it is necessary to develop accurate, reproducible, and noninvasive methods to assess cardiac morphology and function in a serial manner. Ultrasound imaging has been increasingly applied to identify and characterize structural and functional features of different cardiac phenotypes in large animals modeling human diseases [15]. Although the acquisition of mouse echocardiograms is relatively simple, echocardiographic studies are challenging because of the small size, rapid heart beating rate, and orientation of the mouse heart. Recently, a high-resolution echocardiograph has been developed with high-frequency (30–50 MHz) and high-frame rate mechanical probes, allowing an axial resolution of approximately 50 *μ*m at a depth of 5–12 cm [16]. Our laboratory has been among the first to assess cardiac function using the high-frequency echocardiograph (VisualSonics, Inc. Toronto, Canada) on transgenic mice with various cardiomyopathies. In this review, we will summarize the application of the conventional echocardiography, pulsed Doppler, and tissue Doppler imaging for cardiac function assessment in mice and discuss several technical considerations in mouse echocardiography.

## 2. Echocardiography Measurement and Major Parameters

The conventional echocardiography is applied in our laboratory on experimental mice. In addition, pulsed Doppler and tissue Doppler imaging (TDI) are also used to determine hemodynamics and the myocardial motion. B-mode is a two-dimensional real-time image of the heart, which is used to visualize and quantify the anatomical structures. A four-chamber image including left and right atria and ventricles can be obtained from the B-mode. Usually, the operator first locates the structure by B-mode then changes to other modes to measure the cardiac wall motion or blood flow. M-mode is the temporal image of cardiac wall motion along a single ultrasound beam, which enables the quantification of wall thickness and cavity dimensions as well as the movement of myocardium, valves, and vessel walls. Usually, left ventricular end diastolic dimension (LVEDD) and end systolic dimension (LVESD), interventricular septum (IVS), and posterior wall (PW) thickness are recorded with M-mode. Shortening fraction (SF) is calculated from the M-mode LV dimensions using the equation:

(1) $$\text{SF}\;\left(\%\right)=\left[\frac{\left(\text{LVEDD}\;-\;\text{LVESD}\right)}{\text{LVEDD}}\right]\times 100,$$

where SF is an index of systolic left ventricular function. Pulsed Doppler imaging is primarily used for the quantitative measure of blood flow through valves, arteries, or veins, which provides information of both the direction and the velocity of blood flow. Tissue Doppler provides the quantifiable information of myocardial tissue movement, for example, the movement of valves. The major parameters in pulsed Doppler and tissue Doppler measurements will be described in the following sections.

## 3. Assessment of Systolic Function

**Figure 1:**
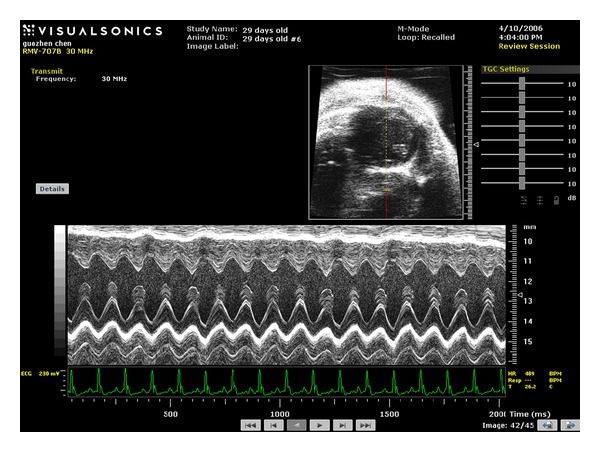
M-mode imaging obtained on a wild type mouse using a 30-MHz transducer.

Left ventricular structure and systolic function is most commonly measured by M-mode in a parasternal short axis view at the papillary muscle level. In order to obtain an anatomically correct parasternal short axis view, operators usually start with parasternal long axis view by placing the transducer vertically to the animal body on the left side of its sternum with the “notch” of transducer pointing to the animal head; and then rotating approximately 30–45 degrees counterclockwise. This view depicts the mid-portion and base of the left ventricle, both leaflets of the mitral valve, the aortic valve and aortic root, the left atrium, and the right ventricle. The parasternal short axis view is obtained by rotating the transducer through 90 degrees clockwise base on the long axis view. Then, the transducer is tilted a little to the apex to visualize the papillary muscles. To ensure all the measure is reliable and repeatable, the short axis view at papillary muscle level is required as a criterion for the imaging used in the later M-mode measure. In other words, the left ventricle is sectioned across the middle of papillary muscles and visualized as a completely round view of it. The left ventricular wall motion along the sampling line is recorded and analyzed in M-mode (Figure 1).

With M-mode imaging of the left ventricle, investigators are able to directly measure the thickness of left ventricular posterior and anterior walls and the interventricular septum. The increase in the wall thickness indicates ventricular hypertrophy while the decrease might be observed in ventricular dilation. Left ventricular mass can be calculated based on the wall thickness. Left ventricular chamber diameter at end-diastole and end-systole is also able to be directly measured. As mentioned above, FS can be calculated from the M-mode LV dimensions. In addition, ejection fraction (EF) is another useful index of systolic function, which can be calculated as well from the M-mode measurements.

**Figure 2:**
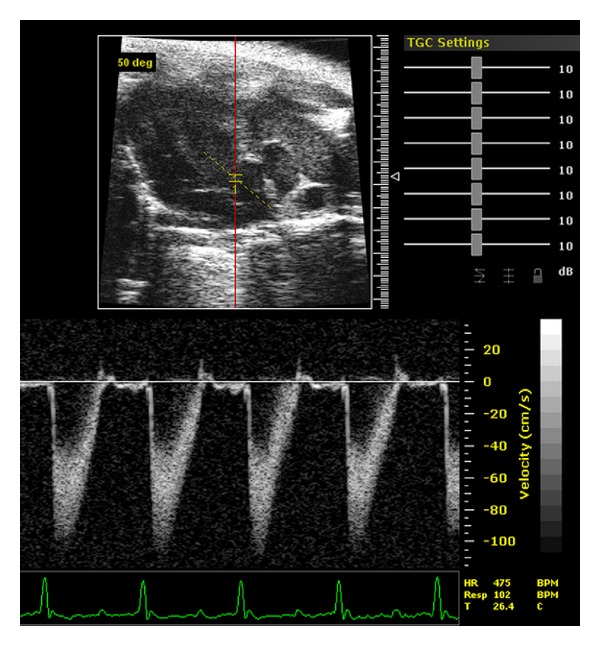
Aortic pulse Doppler tracings obtained on a wild-type mouse.

Pulsed Doppler imaging has been used as well to assess the blood flow through aorta. The quantitative data of the blood flow peak velocity, velocity time integral, the peak, and mean gradient can be measured or calculated, which provide us with useful information on systolic function of experimental animals (Figure 2). Aortic root can be visualized under apical five-chamber view, the fifth chamber is aorta. When the transducer is tilted upward from the apical four-chamber view, aorta will be seen in the middle of the screen (Figure 2).

**An Example of Transgenic Disease Mouse Model with Systolic Dysfunction**

We performed echocardiography studies to measure the cardiac function of cTnT-DE7 transgenic mice (6-month-old) that express an abnormal splice-out of the exon 7-encoded segment in the N-terminal variable region of cardiac troponin T in the hearts. No atrial enlargement, ventricular hypertrophy, or dilation was detected in the hearts of 3-months-old cTnT-DE7 mice, indicating a compensated state. However, left ventricular FS and EF were significantly decreased in 6-month-old cTnT-DE7 mice compared to wild-type controls. Furthermore, the left ventricular outflow tract velocity and gradient were both significantly decreased in the transgenic mouse hearts, indicating decreased systolic function. The impaired systolic heart function *in vivo* without detected changes in diastolic function suggests that cTnT-DE7 primarily reduces contractile function of the cardiac muscle ([17] and unpublished data).

## 4. Assessment of Diastolic Function

In general, cardiac diastolic function can be assessed with conventional echocardiography in B-mode and M-mode. B-mode clearly shows the structure of left ventricle and the dimension of the left ventricle during the systole and diastole. M-mode, furthermore, provides the ability to obtain anatomically correct LV measurements, such as LVESD and LVEDD. Using B-mode and M-mode, we observed a significantly reduced LV end-diastolic dimension in cTnI knockout mice and RCM cTnI transgenic mice, both suffering from a severe diastolic dysfunction [12, 18].

**Figure 3:**
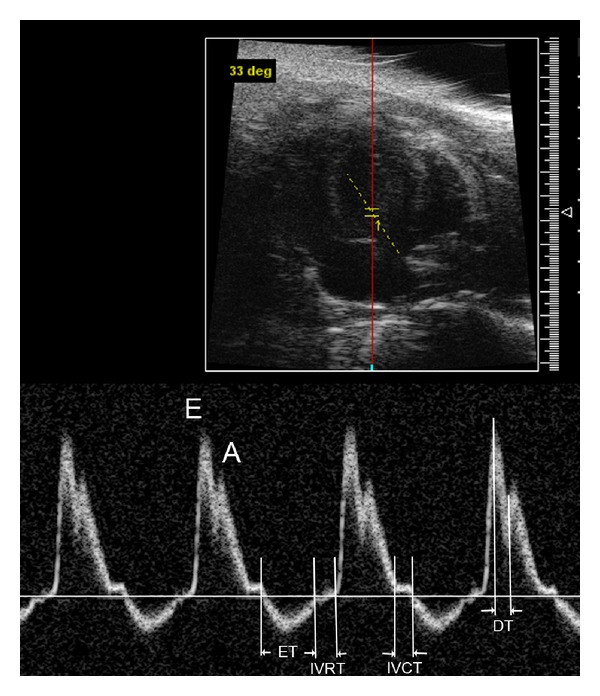
Mitral pulse Doppler tracings obtained on a wild type mouse. E, E wave indicating early ventricular filling; A, A wave indicating late filling caused by atrial contraction; ET, ejection time; IVRT, isovolumetric relaxation time; IVCT, isovolumetric contraction time; DT, deceleration time.

Recently, Doppler echocardiography has emerged as an important clinical tool to provide reliable and useful data for diastolic performance [15, 19]. The noninvasive nature and high-resolution feature of Vevo 770 echocardiography allow us to longitudinally monitor diastolic performance in mice *in vivo*. Using echocardiography to evaluate diastolic function is primarily achieved via pulsed Doppler imaging of transmitral blood flow and tissue Doppler imaging of mitral annulus velocity. They are all accessed under apical four-chamber view, which is obtained from the lower left side of the animal's thorax. This view essentially allows us to look up from the apex towards the base of the heart and visualize the right and left ventricles, and the mitral inflow tract, with the atria at the bottom of the screen (Figure 3). The transducer is angled by 60–70 degrees and placed in a transverse position with the notch facing the left side of the mouse.

As shown in Figure 3, two diastolic waves, E and A, are seen in normal mitral Doppler tracing. The E wave occurs during the rapid filling phase in early diastole and the A wave occurs in late diastole as a result of atrial contraction. Normally, the E wave is higher than the A wave. Clinically useful mitral inflow parameters include the following: early filling peak velocity (E); atrial peak velocity (A); E/A ratio (normally >1); deceleration time (DT) or the interval between the peak of the E wave to the zero baseline. The isovolumetric relaxation time (IVRT) is also measured and is the phase in which all valves are closed and the ventricles relax without any change in volume.

TDI analysis has become an established component of the diagnostic ultrasound examination, which permits an assessment of myocardial motion. Sample volume is measured at the septal side of the mitral annulus. Early (E′) and late (A′) diastolic mitral annulus velocity and the ratio of E′ to A′ are determined. TDI is very useful to distinguish the pseudo-normalization pattern from the normal filling pattern as reported previously [20].

The ratio of early filling transmitral peak velocity (E) over the TDI mitral valve E velocity (E′) has established itself as a reliable guide to elevated pulmonary capillary wedge pressure [21]. Pulmonary capillary wedge pressure (PCWP) provides an indirect estimate of left atrial pressure [22]. Although left ventricular pressure (LAP) can be directly measured by placing a catheter into the left ventricle by feeding it through a peripheral artery, into the aorta, and then into the ventricle, it is not feasible to advance this catheter back into the left atrium. LAP can be measured by placing a special catheter into the right atrium then punching through the interatrial septum; however, for obvious reasons, this is not usually performed because of the damage to the septum. Direct measurement of LAP can be available to human hearts, but it is not applicable to mouse hearts. Therefore, the E/E′ ratio for estimation of LAP in mouse hearts is a very useful tool in the assessment of cardiac diastolic properties.

**An Example of Transgenic Disease Mouse Model with Diastolic Dysfunction**

We applied both pulsed Doppler and TDI to our studies on transgenic mice (from 2–12 months) suffering from restrictive cardiomyopathy due to cTnI mutation [20, 23]. We found an E/A ratio reverse representing a relaxation abnormality in these transgenic mice and restrictive physiology in these mice at the advanced stage [20]. Furthermore, we found that prolongation of IVRT was the earliest sign of impaired relaxation observed in these transgenic mice. This is consistent with previous reports demonstrating that IVRT is the most sensitive Doppler index to detect impaired relaxation because it is first to become abnormal [24, 25].

## 5. Assessment of Coronary Flow and Myocardial Perfusion

**Figure 4:**
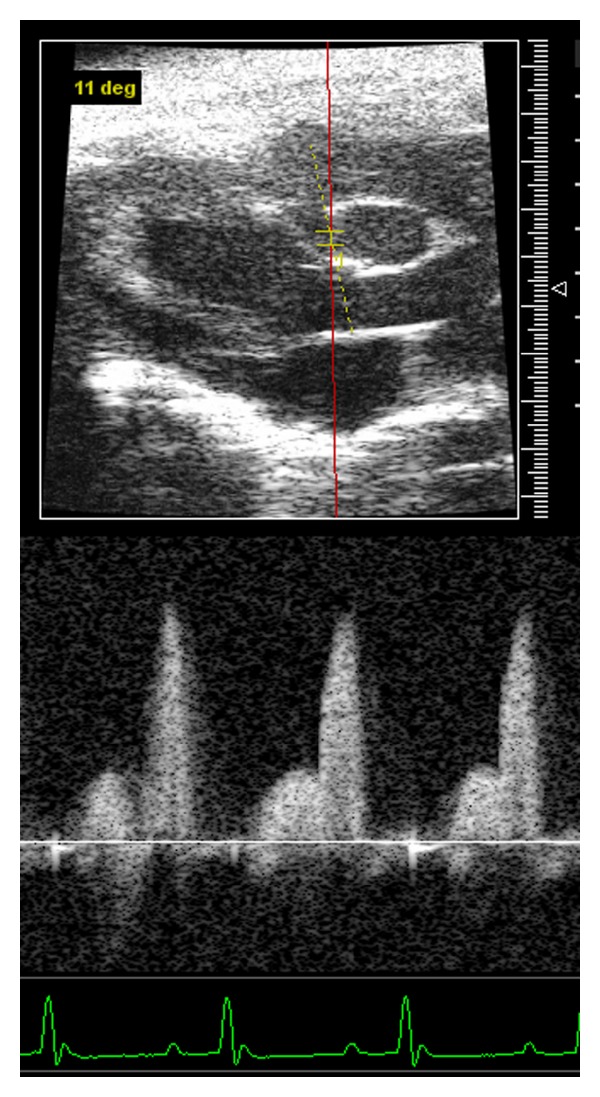
Coronary pulse Doppler tracings obtained on a wild-type mouse.

Pulsed Doppler imaging has been used to assess the blood flow through coronary artery of mouse hearts [26]. From a modified parasternal long axis view, the left coronary artery is visualized stemming from the aorta sinus, traveling between the right ventricle outflow tract and left ventricular anterior wall, and along the left ventricular wall to the distal branch site (Figure 4). Sample volume of pulsed Doppler is placed on the left main coronary artery. Under such condition, Doppler imaging of coronary blood flow consists of two peaks: the preceding low peak representing coronary blood flow in systole and the following high peak representing the coronary perfusion in diastole (Figure 4).

We measured coronary artery blood flow using echocardiography Doppler system in wild-type and transgenic mice (2-month-old) with diastolic dysfunction. Our results showed that a significant decrease of coronary circulation was observed in transgenic mice suffering from diastolic dysfunction (unpublished data). These data indicate that the increased atrial and ventricular end-diastolic pressure observed in diastolic dysfunction can reduce coronary artery blood flow since the coronary blood supply occurs mostly during the diastole. Hartley et al. also reported that a significant reduction of coronary flow reserve (CFR) was observed using noninvasive 20-MHz Doppler ultrasound in mice with pressure overload cardiac hypertrophy [27]. Echocardiographic techniques are, indeed, useful tools for assessing coronary blood flow and myocardial perfusion in mice *in vivo*.

## 6. Examination of Embryonic Mouse Hearts

**Figure 5:**
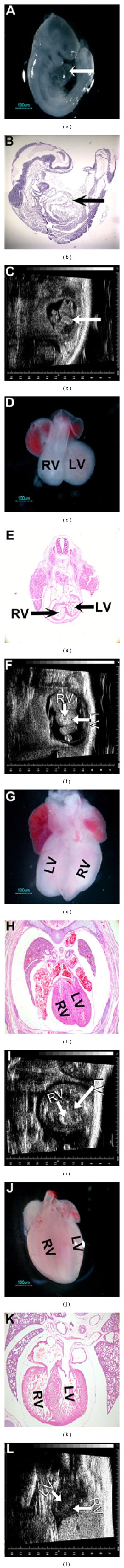
Echocardiographic observations on embryonic mouse heart during the development. Echocardiographic images obtained on embryonic mice from E8.5 to E17.5 ((c), (f), (i), and (l)) are compared with the gross anatomic observations ((a), (d), (g), and (j)) and histological analyses ((b), (e), (h), and (k)) at the same developmental times. The linear heart tubes observed on mouse embryos at day E8.5 are indicated by an arrow ((a), (b), and (c)). RV, right ventricle; LV, left ventricle.

Although technically challenging, evaluation of the cardiac dimension and function of mouse embryos is an invaluable tool to assess the role of genes in the early development of cardiac function [16]. We have examined embryonic mouse hearts using high-resolution echocardiography in B-mode and M-mode. The earliest detection is available on embryonic day 8.5 (E8.5) when the linear heart tube begins to beat. During days E11.5 to 13.5, it is possible to observe atria and ventricles since heart separation is taking place. On day E14.5, it is feasible to measure the ventricular wall thickness and the motion with high-resolution echocardiography in M-mode. From day E14.5 onward, we can examine the four-chamber embryonic heart morphology, analyze the cardiac function, and detect the abnormality in the heart during the development (Figure 5).

## 7. Technical Considerations in Mouse Echocardiography

Cautionary tales of mouse echocardiography have been discussed by several reviews [15, 16, 28]. The common issue is anesthesia and mouse heart rates. Anesthetic agents are generally employed to immobilize and sedate mice for better image acquisition during echocardiographic examination. Heart rate in conscious normal mice is about 600–650 beats/minute. The reduction in heart rate caused by anesthetics in different research models may result in better temporal resolution for improved image visualization, and measurement, but may simultaneously confound the physiological issue in question [15]. In our studies, we initially used a protocol of anesthesia that mice were anesthetized with 5% isoflurane and then maintained at 1.5% isoflorane by a facemask during the whole procedure. It has been reported that using 1.5% isoflurane for anesthesia has minimal effects on cardiac function [29, 30]. However, we observed late that we could reduce the isoflorane concentration to 1% that could maintain the experimental mice immobile with heart rates around 470–500 beats/min during the procedure. However, when the heart rate is over 450 beats/min, it is challenging to obtain the separated E and A waves in pulsed Doppler imaging even using the high-resolution echocardiography. According to our experience, we can differentiate E and A waves in pulsed Doppler imaging from experimental mice when their heart rate is controlled around at 400 beats/min. To avoid possible influence of varied heart rates on the explanations of physiological findings, the key point here is to observe and analyze cardiac function with echocardiography on all experimental mice (including transgenic and wild type control mice) at a similar and comparable heart rate.

In addition, we find that prewarming ultrasound transmission gel and keeping the experimental platform at 37°C are necessary to maintain the stable mouse body temperature and heart rate during the procedure of echocardiography measurements.

## 8. Conclusions

Transgenic mice displaying various cardiomyopathies associated with cardiac sarcomeric protein mutations represent a powerful tool for understanding the mechanisms underlying the initiation and the development of the diseases. High- frequency research echocardiography and pulsed Doppler imaging provide us with a noninvasive and reliable method for the assessment of cardiovascular structural and functional changes in cardiomyopathy mouse models and other murine models. The application of fetal mouse imaging using the high-frequency research echocardiography will open a new way for the studies on fetal cardiac physiology and heart development in mouse models.
